# Acceleration of Aril Cracking by Ethylene in *Torreya grandis* During Nut Maturation

**DOI:** 10.3389/fpls.2021.761139

**Published:** 2021-10-20

**Authors:** Yadi Gao, Yuanyuan Hu, Jiayi Shen, Xuecheng Meng, Jinwei Suo, Zuying Zhang, Lili Song, Jiasheng Wu

**Affiliations:** ^1^State Key Laboratory of Subtropical Silviculture, Zhejiang A&F University, Lin’an City, China; ^2^Sino-Australia Plant Cell Wall Research Centre, School of Forestry and Biotechnology, Zhejiang A&F University, Lin’an City, China

**Keywords:** *Torreya grandis*, aril cracking, ethylene, cellular wall polysaccharides, RNA-seq

## Abstract

Torreya *grandis* ‘Merrillii’ is a famous nut with great nutritional value and high medicinal value. Aril cracking is an important process for seed dispersal, which is also an indicator of seed maturation. However, the cracking mechanism of *T. grandis* aril during the maturation stage remains largely unknown. Here, we provided a comprehensive view of the physiological and molecular levels of aril cracking in *T. grandis* by systematically analyzing its anatomical structure, physiological parameters, and transcriptomic response during the cracking process. These results showed that the length of both epidermal and parenchymatous cell layers significantly increased from 133 to 144 days after seed protrusion (DASP), followed by a clear separation between parenchymatous cell layers and kernel, which was accompanied by a breakage between epidermal and parenchymatous cell layers. Moreover, analyses of cell wall composition showed that a significant degradation of cellular wall polysaccharides occurred during aril cracking. To examine the global gene expression changes in arils during the cracking process, the transcriptomes (96 and 141 DASP) were analyzed. KEGG pathway analysis of DEGs revealed that 4 of the top 10 enriched pathways were involved in cell wall modification and 2 pathways were related to ethylene biosynthesis and ethylene signal transduction. Furthermore, combining the analysis results of co-expression networks between different transcription factors, cell wall modification genes, and exogenous ethylene treatments suggested that the ethylene signal transcription factors (*ERF11* and *ERF1A*) were involved in aril cracking of *T. grandis* by regulation of *EXP* and *PME*. Our findings provided new insights into the aril cracking trait in *T. grandis*.

## Introduction

Fruit cracking often occurs under unsuitable environmental conditions and can easily cause adverse impacts to fruit marketability, such as destroying the appearance and increasing the susceptibility of fruit to pathogen infections (Ramteke et al., 2017; Jiang F. et al., 2019). Unlike fruits, pericarp dehiscence is an important and necessary biological process for kernel extraction of nut species, which can also aid in seed dispersal. Significant progress has been made in understanding the mechanisms of fruit cracking in tomato, sweet cherries, apple, and litchi among many others (Domínguez et al., 2012; Khadivi-Khub, 2015; Weksler et al., 2015; Correia et al., 2018; Ginzberg and Stern, 2019; Wang et al., 2019a). However, our understanding of the mechanisms that underlie the cracking process of nuts is surprisingly limited.

Pericarp softening is an undesirable feature during the fruit cracking process, resulting in a loss of firmness, which is mainly caused by changes in cell wall structure and composition, such as cellulose, hemicelluloses, and pectin (Brummell and Harpster, 2001; Brummell, 2006; Bennett and Labavitch, 2008). Previous studies showed that fruit cracking was accompanied by changes in cellular wall polysaccharide content, especially a continuous increase in water-soluble pectin (WSP) arising from chelator soluble pectin (CSP), sodium carbonate-soluble pectin (SSP), and hemicellulose (Chen et al., 2016; Jiang F. et al., 2019; Schumann et al., 2020). During ripening, several hydrolytic enzymes and proteins located at the cell wall, including pectin methylesterase (PME), polygalacturonase (PG), pectate lyases (PL), β-galactosidase (β-gal), β-glucosidase (β-Glu), and expansin protein (EXP), cooperatively disassemble wall polysaccharide networks, thereby contributing to the softening and cracking of fruit (Carpita et al., 2001; Marin-Rodriguez, 2002; Ruiz-May and Rose, 2013). Numerous reports have shown that genes, such as *PME*, *PG*, *EXPs*, β*-gal*, and *PL* are associated with fruit cracking (Schuch et al., 1991; Moctezuma et al., 2003; Huang et al., 2006; Lu et al., 2006; Wang et al., 2006; Kasai et al., 2008). Transgenic suppression of PME activity in tomato caused them to remain firm during ripening (Phan et al., 2007). The transcription level and enzyme activity of MdPG must be correlated with loss of fruit firmness (Wakasa et al., 2006; Mann et al., 2008), whereas inhibition of *LePG* in tomato fruit slightly reduces its cracking rate (Schuch et al., 1991). Simultaneous suppression of *SlPG* and *SlEXP1* in tomato ripening reduced the cracking rate by 12% by decreasing cell wall disassembly (Jiang F. et al., 2019). Inhibition of *EXP1* and β*-gal* 4 (TBG4) significantly increased tomato fruit firmness compared to the control fruit (Brummell et al., 1999; Smith et al., 2002). Therefore, cell wall metabolism is a key factor leading to fruit cracking.

Transcriptional regulation of cell wall-modifying genes through transcription factors (TFs) is an important controlling mechanism for fruit ripening. Previous research showed that fruit ripening and cracking were tightly connected with rapidly increased ethylene emission (Wakasa et al., 2006; Wang et al., 2009). *ERFs*, signal factors that bridge the internal and external signal and ethylene response, have been extensively used in fruit growth and development (Yin et al., 2012). It has been well documented that ERF transcription factors may play roles in cell wall modification during fruit ripening or the cracking stage (Taylor-Teeples et al., 2015; Lee et al., 2016; Sakamoto et al., 2018; Wang et al., 2019b; Wessels et al., 2019). ERF proteins can function as transcriptional activators or repressors, depending on their sequence features (Fu et al., 2016). Chapman et al. (2012) reported that one *ERF* and three *PME* genes were identified as candidate genes that mostly accounted for tomato firmness. In contrast, *PpeERF2* in peach and *CpERF9* in papaya were identified as transcriptional repressors of the cell wall modification genes to control fruit ripening and softening by directly binding to the promoters of *PpePG1* or *CpPG5* and *CpPME1/2*, respectively (Fu et al., 2016; Wang et al., 2019c). Therefore, the exploration of the relevant transcriptional factor for fruit cracking is a prerequisite to successfully underlying its cracking mechanism.

*Torreya grandis* cv. ‘Merrillii’ belonging to the Taxaceae family (Torreya subgenus), is an evergreen, coniferous, subtropical nut with great nutritional value that is rich in oil (about 55%) and unsaturated fatty acids (about 80%) and is popularly consumed in China (Li and Dai, 2007). Moreover, *T. grandis* is also exploited for its high medicinal value since it has multiple healthy bioactive compounds, such as sciadonic acid, squalene, β-sitosterol and tocopherol (Wu et al., 2018; Lou et al., 2019; Suo et al., 2019). Similar to other nut species, a longitudinal crack in the aril of *T. grandis* is an important biological process for kernel extraction, which is an indicator of nut maturation. Our previous study showed that the kernels from the cracked *T. grandis* were superior in quality to those without cracking (Ye et al., 2017). In practice, since the *T. grandis* nut does not develop uniformly, nuts are harvested manually from different stages of the same tree at the same time (many non-cracked nuts were also harvested), leading to the increasing costs and processing time for kernel extraction. Therefore, it is of great significance to reveal the physiological and molecular mechanisms underlying seed cracking in *T. grandis* and seek a technology with an increased cracking rate that can shorten the kernel extraction time and achieve high-quality kernels to meet the increasing demands for *T. grandis* nuts.

In this study, our aim was to obtain a comprehensive view of the physiological and molecular levels of aril cracking in *T. grandis* ‘Merrillii.’ First, alterations in anatomical structure, firmness and cell wall fractions were investigated during the *T. grandis* nut cracking stage to identify which cell wall fractions accounted for cracking. Transcriptome analysis of aril at two different development stages was performed to identify the functional genes for nut cracking. Then, the candidate transcriptional factors regulating seed cracking were identified by co-expression analysis with cell wall degradation-related genes and TFs were also investigated using epidermal and parenchymatous cell layers from 113 to 154 days after seed protrusion (DASP) to determine whether these genes were induced by 1-methylcyclopropene (1-MCP) or ethylene. These findings reported here can increase our understanding of the transcriptional regulatory mechanisms of nut cracking, which will help kernel extraction for seed dispersal and enhance kernel quality.

## Materials and Methods

### Plant Material and Growing Conditions

*Torreya grandis* cv ‘Merrillii’ grafting trees were grown at Yuqian Town, Lin’an District, Hangzhou City, Zhejiang Province, China (30°14′N, 119°42′E). These trees were grafted with a 1-year-old *T. grandis* cv. ‘Merrillii’ scion on a 2-year-old *T. grandis* Fort. Ex Lindl. root stock in 2001, and they have borne seeds since 2009. Trees used for sample collection were maintained with standard horticultural practices and methods for disease and insect control (Hu et al., 2018). The development of *T. grandis* seeds lasts for 17 months, from fertilization through the rapid longitudinal and transverse elongation of seeds and aril cracking to complete the development. Typically, *T. grandis* seeds are manually harvested at “white dew” (name of season in old calendar, corresponding to early September) with a 20% cracking rate. Since the seeds of *T. grandis* do not develop uniformly, each of the seeds to be sampled was labeled with a plastic tag on the day when the seed scale was breached. In the present study, the seeds began to stick out from the seed scale on April 15th in 2019. White dew occurred on September 8th. The aril material was collected at four different developmental stages: 113, 133, 144, and 154 days after the seeds protrusion (DASP). Twenty seeds from each tree and each stage were mixed into one biological replicate for further analysis, and in total, three biological replicates were collected for each stage. The total aril were separated into two parts, that is, the epidermal cell layers (ECL) and the parenchymatous cell layers (PCL). Next, the total aril, ECL and PCL were sampled during the different developmental stages, immediately frozen in liquid nitrogen, and stored at −80°C until use for cell wall component, transcriptome, and qPCR analyses.

A separate experiment with exogenous ethephon and ethylene inhibitor (1-methylcyclopropene, 1-MCP) treatments was carried out in 2020 with non-cracked seeds (for details, see Section “Quantitative RT-qPCR”).

### Measurement of Seed Aril Firmness

Seed firmness was measured using an FHR-5 Analyzer (Japan) with a meter probe 12 mm in diameter and a penetration depth of 3 mm. The firmness of each seed aril was averaged from four measurements of 90° apart at the seed equator (10 individual seeds per replicate at each stage). Firmness was expressed as Newtons (N).

### Anatomical Structure of Seed Aril

Aril samples were taken from seeds at 133, 144, and 154 DASP for the analysis of light micrograph cross-sections. Fresh aril samples were fixed in formalin-acetic acid alcohol (FAA), dehydrated in an ethanol series, and embedded in paraffin. Transverse sections (8 μm) were cut with an automatic microtome (Leica RM2265, German). After double-staining with Safranin-Fast Green, photomicrographs were taken with an optical microscope (OLYMPIC BX60, Japan).

### Determination of Cell Wall Components

Extraction and determination of pectin and cellulose content were measured according to the method by Vicente et al. (2007). Seed aril samples (1 g) were ground with liquid nitrogen and homogenized for 2 min in 10 mL ethanol (95%, v:v). The homogenate was then boiled for 1 h, cooled, and filtered through a glass filter. The insoluble residue was then washed with 95% ethanol and refiltered. Then, the residue was extracted for 15 min with 10 mL chloroform: methanol (1:1, v:v), filtered, and rewashed with the same solvent mixture, followed by a final wash with 10 mL of acetone. The alcohol-insoluble residue (AIR) was dried at 37°C for 48 h to a constant weight, which was identified as the total cell wall fraction of ground tissue.

Fractions enriched for pectin polymers of the isolated cell wall preparations were stepwise extracted from AIR. AIR (0.1 g) was suspended and stirred for 12 h at 28°C in 10 mL distilled water, then centrifuged at 10,000 *g* for 30 min at 20°C. The supernatant was removed and kept separate. The remaining pellet was re-suspended and stirred in distilled water twice for extraction. Then, the three supernatants were combined as water-soluble pectin (WSP, fraction mainly pectin with no strong bonds to the rest of the cell wall). The residue was then extracted in 10 mL 50 mM cyclohexane-*trans*-1,2-diamine tetra-acetate (CDTA; pH 6.5) three times to obtain the chelator soluble pectin (CSP, the faction mainly pectin that was ionically bound into the wall via linkages to Ca^2+^). The residue, after CSP extraction, was further extracted with 10 mL of 50 mM Na_2_CO_3_ (containing 20 mM NaBH_4_), three times, to obtain sodium carbonate-soluble pectin (SSP, the faction containing pectin covalently bound by ester linkages into the cell wall).

Hemicellulose was extracted in 4% KOH containing 20 mM NaBH_4_ for 2 h at 28°C. The extraction process was repeated four times. The residue was recovered by filtration, washed with water, dried, and reweighed. Weight loss was attributed to the 4% KOH-soluble hemicellulose fraction (4KSF). The tight bound hemicellulose fraction (24% KOH-soluble fraction, 24KSF) was obtained from the residue by extraction with 24% KOH containing 20 mM NaBH_4_. The residue after KOH extraction was used for cellulose determination following the protocols of Vicente et al. (2007).

### Ethylene Production of Seed Aril

Before ethylene measurement, 12 whole seeds (with aril) and 12 kernels (removed the aril) per replicate were weighted and sealed individually in air-tight jars (0.25 L) for 8 h at 25°C. Head-space gas (1 mL) was removed from each jar using a syringe, and ethylene was measured by gas chromatography using a gas chromatograph (Thermo TRACE 1300) fitted with aflame ionization detector and a TG-BOND column (30 m × 0.53 mm × 20 μm). The injector, detector, and oven temperatures were 200°C, 250°C, and 100°C, respectively.

### Complementary cDNA Library Construction and Sequencing

Total RNA was extracted from aril at each stage using the RNA prep Pure Plant Kit (DP441, Tiangen), after which the RNA samples were combined for sequencing. mRNA was purified from total RNA using polyA oligo-attached magnetic beads and segregated into 200–300 bp fragments. First-strand DNA was synthesized from 6-base random primers and reverse transcriptase using RNA as a template, and second-strand cDNA was synthesized using the first-strand cDNA as a template. In second-strand cDNA synthesis, the base T was replaced by U to generate a chain-specific library. RNA-Seq was performed using chain-specific kits, and chain-specific libraries were used to determine the transcriptional direction of sense and antisense strands to increase the accuracy of subsequent gene function annotation and gene expression analysis. After library construction, library fragments were enriched by PCR amplification, and selection was based on fragment size, resulting in a 300–400 bp library. The library was tested using an Agilent 2100 Bioanalyzer (Agilent, Palo Alto, CA, United States), and both the total and effective concentrations of the library were tested. The datasets are deposited in the NCBI database with the accession number SRP338664.

Based on the effective concentration and the amount of data required, libraries containing different index sequences (samples plus different index sequences, and difference data of samples according to index) were mixed proportionally. The mixed library was diluted to 2 nM and alkaline degeneration was performed to generate a single-chain library. Subsequently, 150 bp pair-end reads were collected in the library using an Illumina NextSeq sequencing platform to carry out next-generation sequencing (NGS) at Personalbio (Personalbio, Shanghai, China). For each treatment at each time point, the sample was sequenced three times as three technical replicates.

Clean data were obtained from the raw data by removing adapters, ploy-N sequences, and poor-quality data. Then, Q20, Q30, and GC content, as well as the sequence duplication level of the clean data were calculated, serving as the basis for all downstream analyses. Cured reads were assembled using the Trinity program. The full-length transcripts were compared against public databases: the National Center for Biotechnology Information (NCBI), Non-redundant (Nr) and Nucleotide (Nt) databases, Swiss-Prot, Kyoto Encyclopedia of Genes and Genomes (KEGG^1^), Cluster of Orthologous Groups of proteins (COG), and TrEMBL using BLASTX with an *E*-value of ≤10^–5^. Gene ontologies (GOs) were assigned to each full-length transcript using Blast2GO.

### Differential Expression Gene Analysis

Differential expression analysis using data collected at different developmental stages was performed using the DESeq package. Genes with an adjusted *p*-value < 0.05 (*q*-value < 0.05) and | log2FoldChange| > 1 in DESeq analysis were assigned as DEGs. In GO enrichment analysis, only categories with a *p*-value < 0.05 were considered enriched in the network. The KEGG database was used to indicate the location of the DEGs in the different pathways. Pathways with *P*-values < 0.05 were considered statistically significant.

### Effects of Ethephon and 1-Methylcyclopropene on Aril Cracking in *T. grandis*

Non-cracking *T. grandis* seeds were harvested by hand on September 8th in 2020. The seeds were free of mechanical injury, insects, and diseases and were selected and divided into three groups. Our treatments included (1) control (CK), *T. grandis* seeds placed into plastic boxes after spraying with 5 mL H_2_O and covered with a plastic bag, then kept at room temperature (RT, 25 ± 1°C; (2) 1-methylcyclopropene (1-MCP), *T. grandis* seeds placed inside a sealed container with 200 nL L^–1^ 1-MCP after spraying with the same volume of H_2_O as in the control, fumigated for 24 h at RT, then ventilated, and held under ambient conditions at RT; and (3) ethephon (ETH), *T. grandis* seeds treated with 5 mL 3,000 μL L^–1^ ethephon solution, fumigated for 24 h at RT, then ventilated, and held under ambient conditions at RT. All the seeds were sampled and measured at 9 days of treatment after three applications. Aril firmness and ethylene emission of the seeds were measured at 9 days of treatments. The aril were sectioned, immediately frozen in liquid nitrogen and stored at −80°C for further real-time PCR analysis.

### Quantitative Quantitative Real-Time PCR

RNA was extracted from the aril as described above. First-strand cDNA were synthesized from 1 μg of total RNA using the PrimeScript RT Master Mix (Takara) according to the manufacturer’s instructions. Gene expression levels were determined by qRT-PCR using ChamQ SYBR Green qPCR Master Mix (Vazyme) on a C1000 Touch Thermal Cycler (Bio-Rad). The primers were designed using Prime3 online software^2^ and the specificity of primers was confirmed by both melting curves and product sequencing before use. The relative expression profiles were analyzed and relative expression level of the genes was calculated using the 2^–ΔΔ*Ct*^ method and using expression of the *TgActin* as the internal control (Lou et al., 2019; Ding et al., 2020). The reaction conditions were 45 cycles of 95°C for 10 s, 57°C for 10 s, and 72°C for 20 s. The experiment was performed with three biological and technical replications.

### Statistical Analysis

Data were subjected to analysis of variance (ANOVA) using SPSS statistical software (16.0, IBM, New York, United States). Duncan’s multiple range test was used to compare the means in aril among different sampling occasions (from 113 to 154 DASP). The data are expressed as the mean ± standard deviation (SD). Differences at *P* < 0.05 were considered significant. The difference in the average values between the epidermal cell layers (ECL) and parenchymatous cell layers (PCL) of aril was tested by the independent-sample *t*-test: ^∗^*P* < 0.05, ^∗∗^*P* < 0.01, ^∗∗∗^*P* < 0.001; ^*ns*^*P* > 0.05. The relationships between cell wall modification genes and ethylene signal transcription factors were determined using correlation analysis.

## Results

### Changes in Aril Structure During the Cracking Process

**FIGURE 1:**
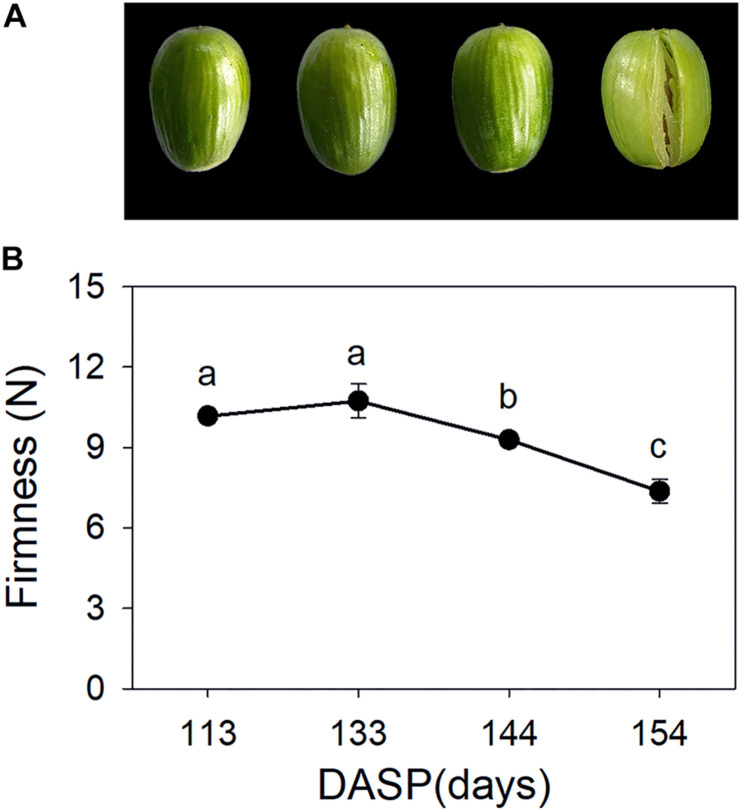
Changes in aril appearance and firmness in *Torreya grandis* during nut maturation. **(A)** Appearance of whole nuts; **(B)** aril firmness. Error bars represent standard error base on three biological replicates. Different lowercase letters within a row indicate a significant difference at different growth stages by Duncan’s multiple range test (*P* < 0.05). *N* = 3.

**FIGURE 2:**
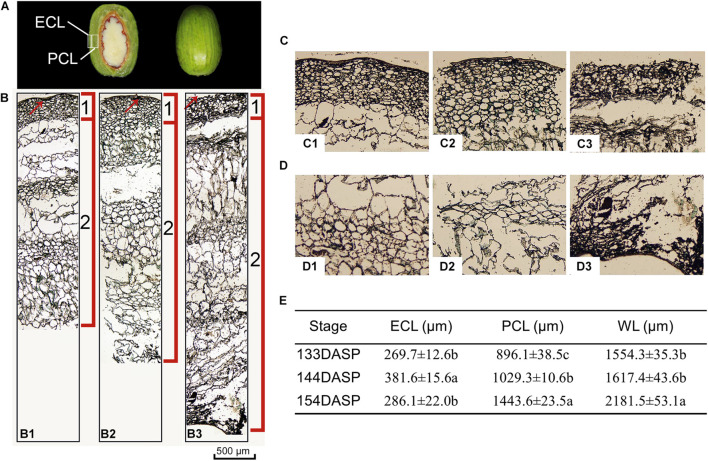
Changes in light micrograph longitudinal sections of epidermal and parenchymatous aril cells in *Torreya grandis* during nut maturation. **(A)** Appearance of ECL (epidermal cell layer) and PCL (parenchymatous cell layer) in the aril. **(B)** Light micrograph longitudinal sections of whole aril. (B1–B3) Anatomical structural of the whole aril at 133, 144, and 154 days after seed protrusion (DASP), respectively; 1 indicates the epidermal cell layer; 2 indicates the parenchymatous cell layer; the red arrow indicates the cuticle. **(C)** Light micrograph of epidermal aril cells. (C1–C3) Anatomical structure of the epidermal cells layer at 133, 144, and 154 DASP, respectively. **(D)** Light micrograph of parenchymatous aril cells. (D1–D3) Anatomical structure of the parenchymatous cell layer at 133, 144, and 154 DASP, respectively. **(E)** Thickness of the ECL, PCL, and WL (whole layer). Different lowercase letters within a row indicate a significant difference at different growth stages by Duncan’s multiple range test (*P* < 0.05). *N* = 3.

*Torreya grandis* seeds are composed of the aril and the kernel. As shown in Figure 1A, aril firmness remained stable from 113 to 133 DASP; however, it decreased to a significantly lower level at 144 DASP than at 133 DASP (Figure 1B). The dynamic anatomical structure of aril during different stages of the cracking process was also evaluated (Figure 2A,B). No cracks in the surface cuticle were found at 133 DASP using paraffin sectioning, and cracks appeared at 144 DASP (Figure 2B1–B3). The epidermal and parenchymatous cells were densely arranged, with small intercellular spaces and a continuous distribution at 133 DASP. However, the cell volume of both epidermal and the parenchymatous cell layers significantly increased at 144 DASP compared with 133 DASP, and the cells at the edge began to degrade and separate (Figure 2B2,C2). For 154 DASP, breakages in the cuticle on the surface gradually became bigger, and larger spaces were observed between the epidermal and parenchymatous cell layers (Figure 2C2,C3). In addition, obvious separation layers were observed between the parenchymatous cell layer and shell as evidence of total cracking at 154 DASP (Figure 2D). The thickness of the epidermal and parenchymatous cell layers increased by 111.8 and 389.2 μm, respectively, from 144 to 154 DASP (Figure 2E).

### Changes in Cell Wall Components of the Aril During the Cracking Process

**FIGURE 3:**
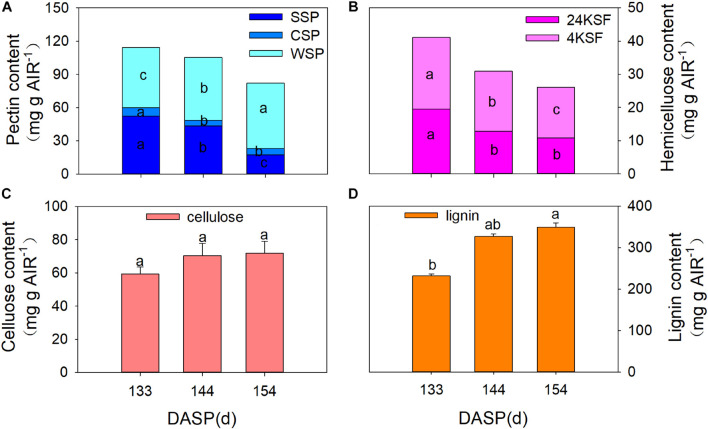
Changes in aril cell wall components in *Torreya grandis* during seed development stages. **(A)** WSP, water solubilized pectin, CSP, CDTA solubilized; SSP, Na2CO3 solubilized. **(B)** 24KSF, 24% KOH-soluble hemicellulose fraction; 4KSF, 4% KOH-soluble hemicellulose fraction. **(C)** Cellulose and **(D)** lignin. Different lowercase letters indicate a significant difference at different growth stages by Duncan’s multiple range test (*P* < 0.05). *N* = 3.

Numerous studies have reported that pericarp or aril breakdown in fruits is a result of cell wall modification, especially cell wall polysaccharides (Brummell, 2006; Bennett and Labavitch, 2008; Billy et al., 2008). Therefore, changes in the cell wall composition of the aril were also investigated during seed maturation in the present study. SSP, CSP, and total pectin showed a pronounced decrease from 133 to 154 DASP. The SSP fraction significantly decreased by 17.3 and 60.1% from 133 to 144 DASP and 144 to 154 DASP, respectively (Figure 3A). The CSP significantly decreased by 32.1% at 144 DASP in comparison to 133 DASP, then remained stable from 144 to 154 DASP. However, the WSP fraction showed a pronounced increase during 133 to 154 DASP. Both tightly bound (24% KOH-soluble fraction, 24KSF) and loosely bound hemicelluloses (4% KOH-soluble fraction, 4KSF) gradually decreased with *T. grandis* ripening (Figure 3B). The 24KSF significantly decreased by 34.1% at 144 DASP compared to 133 DASP, followed by a stable trend. A significant reduction of 4KSF was 16.6 and 15.4% from 133 to 144 DASP and 144 to 154 DASP, respectively. The significantly increased WSP accompanied by the significant reduction of 4KSF and 12KSF coincided with the decreased aril firmness during seed maturation. The cellulose content remained stable during ontogeny (Figure 3C). The lignin content significantly increased from 133 to 144 DASP and still increased until 154 DASP (Figure 3D), which was consistent with our optical microscope observation of a clear lignification area at the site of separation between parenchymatous cell layer and shells.

### Transcriptome Analysis Overview

To obtain a better understanding of the molecular mechanisms underlying the cracking process of *T. grandis* during the seed development stage, transcriptome analysis was performed using RNA-Seq. A total of six RNA-Seq libraries were constructed and sequenced. These libraries were from aril samples at two time points (96 and 141 DASP), with three biological replicates for each time point. From 96 and 141 DASP libraries, 71.40 and 74.29 million raw sequence reads, respectively, were generated (Supplementary Table 1). After removing low-quality reads and adaptor sequences, 71.16 and 73.99 million clean reads were obtained with 97.32–97.85% Q20 bases and 93.51–94.70% Q30 bases, respectively.

Approximately 78.41% (95,027/121,185) unique sequences were annotated based on BLASTx (cutoff *E*-value 10^–5^) searches of five public databases: NCBI non-redundant (NR) database, SwissProt protein (Swissprot) database, Kyoto Encyclopedia of Genes and Genomes (KEGG) database, Non-supervised Orthologous Groups (NOG) database and Gene Ontology (GO) database (Supplementary Figure 1). Based on the *E*-value distribution, 42.64% of the unigenes showed very strong homology (*E*-value < 1e^–100^) to available plant sequences. Supplementary Figure 1C shows that approximately 53,532 unigenes were annotated to five top-hit species, including *Picea sitchensis*, *Amborella trichopoda*, *Nelumbo nucifera*, *Vitis vinifera*, and *Elaeis guineensis* (Supplementary Figure 1).

### Functional Classification of Identified Differential Expression Gene

Data from the latter time point were compared with the former (96 vs. 141 DASP) to identify DEGs. This analysis resulted in the identification of a total of 18,380 genes that showed differential expression patterns between 96 and 141 DASP (96 vs. 141 DASP), and 9,155 and 9,225 genes were up- and downregulated, respectively (Supplementary Figure 2).

**FIGURE 4:**
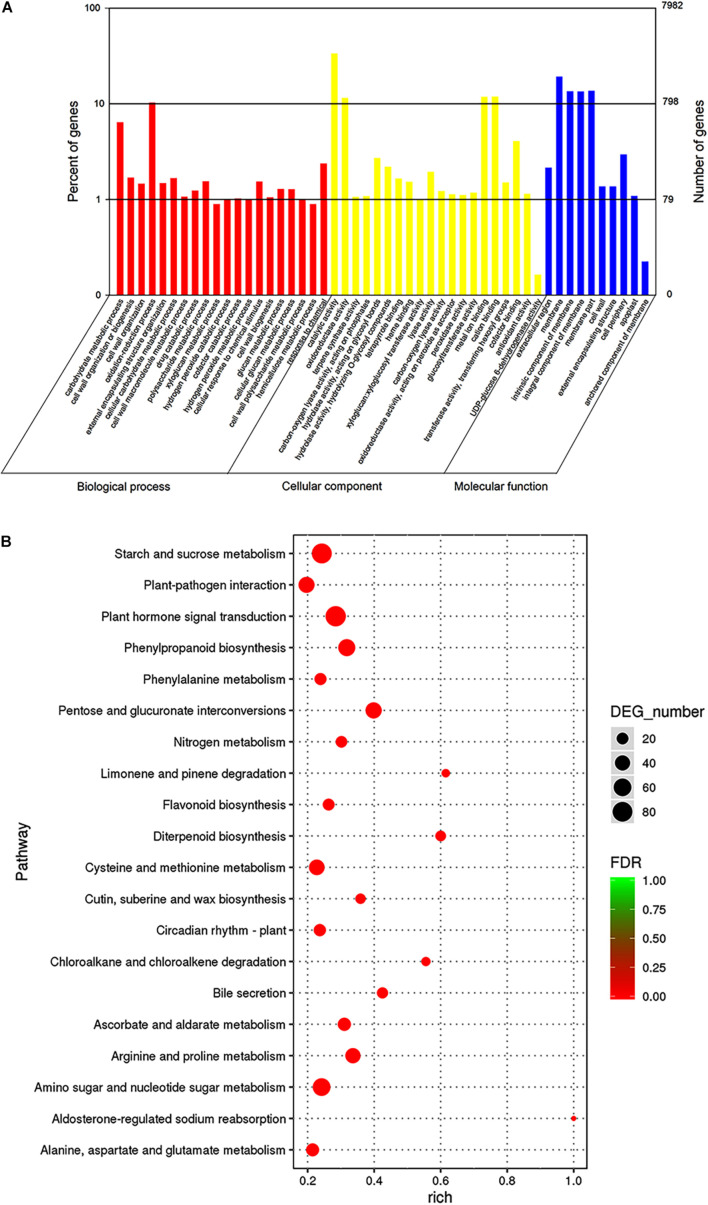
Gene ontologies classification **(A)** and KEGG enrichment **(B)** of DEGs between 96 and 141 DASP at the transcript level. **(A)** GO classifications, the *x*-axis indicates the GO term, and the *y*-axis indicates the number of DEGs. **(B)** KEGG enrichment, the dot size indicates the number of DEGs in certain pathways. A larger size represents more DEGs. The color shade of the point represents the change in false discovery rate (FDR). A smaller FDR indicates more statistical significance. *N* = 3.

To investigate the trends in gene function and enrichment for DEGs, GO analysis was performed. DEGs (7,982) were classified into three main categories: “biological process,” “cellular component,” and “molecular function” (Figure 4A). Most DEGs were enriched in “biological process” GO term, such as the “oxidation-reduction process,” “carbohydrate metabolic process,” “cell wall organization or biogenesis,” and “response to chemicals.” The major groups within the “cellular component” category were “catalytic activity” (4,578 unigenes, 57.35%), “oxidoreductase activity” (1,227 unigenes, 15.37%), “metal ion binding” (1,320 unigenes, 16.54%) and “cation binding” (1,332 unigenes, 16.69%). The majority of DEGs belonged to the membrane in “molecular function” category, followed by “cell periphery,” “cell wall,” and “external encapsulating structure” (5.24, 2.23, and 2.23%, respectively).

To better understand the mechanism of seed cracking, the DEGs were annotated using the KEGG database. The top 20 categories are shown in Figure 4B. Most of the isoforms were included in the “metabolism” category (Supplementary Table 3), and the top 5 enriched pathways related to “metabolism” were pentose and glucuronate interconversions (ko00040), phenylpropanoid biosynthesis (ko00940), starch and sucrose metabolism (ko00500), arginine and proline metabolism (ko00330), and amino sugar and nucleotide sugar metabolism (ko00520). Most of these pathways can be integrated into the cell wall metabolism network. In addition, cutin, suberine and wax biosynthesis (ko00073) was enriched in the first 15 pathways, indicating that it played an important role in the cracking process. The first top pathway “Plant hormone signal transduction” was included in the “environmental information procession” category.

### Identification of Differential Expression Genes Associated With Candidate Pathways During Aril Cracking

A total of 47 DEGs were annotated in the pentose and glucuronate interconversions, including *PME*, *PL* and *UDP-glucose 6-dehydrogenase*, which were upregulated at 141 DASP compared with 96 DASP (Supplementary Tables 2, 3). The phenylpropanoid pathway-related genes *peroxidase* (*POD*), *4-coumarate-COA-ligase* (*4CL*), and β*-glucosidase* (β*-Glu*) were upregulated at 141 DASP compared with 96 DASP (Supplementary Tables 2, 3). A total of 81 DEGs were enriched for the term starch and sucrose metabolism, which contained *PME*, *PG*, and β*-Glu* and were significantly upregulated at 141 DASP compared to 96 DASP (Supplementary Tables 2, 3).

**FIGURE 5:**
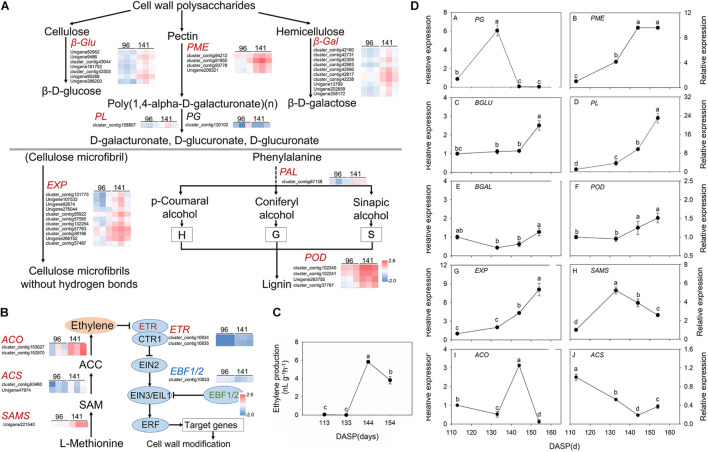
Pathway of cell wall modification, ethylene metabolism, ethylene production and expression of the main genes involved in these pathways in *Torreya grandis* during nut maturation. **(A)** Heat map of the differentially expressed transcripts involved in cell wall modification. **(B)** Heat map of the differentially expressed transcripts involved in the ethylene metabolic pathway. **(C)** Ethylene production of the aril during nut maturation. **(D)** Quantitative real-time PCR (RT-qPCR) of the main genes. Genes in red are significantly upregulated, genes in blue are significantly downregulated, and genes in black have no significant changes. The standard is | log2FoldChange| > 1.5, *p*-value < 0.05, FPKM > 10. The expression level was homogenized by log10. PME, pectin methylesterase; PG, polygalacturonases; PL, pectate lyases; β-gal, β-galactosidase; β-Glu, β-glucosidase; EXPs, expansion protein; POD, peroxidase; SAMS, *S*-adenosylmethionine synthase; ACS, 1-aminocyclopropane-1-carboxylate synthase; ACO, 1-aminocyclopropane-1-carboxylate oxidase. Different lowercase letters indicate a significant difference at different growth stages by Duncan’s multiple range test (*P* < 0.05). *N* = 3.

According to GO and KEGG analysis, most DEGs were significantly enriched in cell wall modification pathways. Then, a pathway diagram of cell wall modifications during seed cracking was mapped based on DEGs and previous studies (Figure 5A). An enrichment of cell wall polysaccharide metabolism-related unigenes was also observed, which participated in pectin degradation, including three kinds of genes: *PME*, *PG*, and *PL*. Five *PME* genes (cluster_contig 91920, cluster_contig 93778, cluster_contg 91975, cluster_contig 92543, and Unigene 206321) and three *PL* genes (Unigene 204828, cluster_contig 158807 and cluster_contig 11813) were upregulated and one *PG* (cluster_contig 104969) and one *PL* (cluster_contig 174686) were downregulated at 141 DASP compared to 96 DASP. Four unigenes encoding β*-Glu* (Unigene 82962, Unigene 285144, cluster_contig 158581, and Unigene 90288) involved in cellulose degradation were also upregulated at 144 DASP compared to 96 DASP. Seven unigenes encoding β*-Gal* (cluster_contig 42160, cluster_contig 42731, cluster_contig 42309, cluster_contig 42663, cluster_contig 42596, cluster_contig 42817, cluster_contig 42238, Unigene 13799, Unigene 202659, and Unigene 258172) involved in hemicellulosic compounds were also up- or downregulated at 144 DASP compared to 96 DASP. As the anatomical structure showed, both epidermal and parenchymatous cell layers expanded significantly from 113 to 144 DASP, which was consistent with the 19 *EXPs* being upregulated at 141 DASP compared to 96 DASP (Figure 5A), resulting in enhancing cell wall extensibility and inducing cell expansion (Figures 2B,E). Meanwhile, one *PAL* (cluster_contig67138) and seven *PODs* (cluster_contig 101423, cluster_contig 102240, cluster_contig 163476, Unigene 118590, Unigene 171186, Unigene 265816, and cluster_contig 39638) were upregulated at 141 DASP compared to 96 DASP (Figure 5A). Numerous studies showed that the malleability of the cell wall were decreased by POD activating phenolic groups (Lin and Kao, 2001; Andrews et al., 2002; Cordoba-Pedregosa et al., 2003). Furthermore, many genes associated with wax synthesis, including *CYP86*, *ACE/HTH*, *CER1*, and *FAR*, were significantly downregulated at 141 DASP compared to 96 DASP (Supplementary Figure 3), which coincided with the occurrence of cracks in the cuticle (Figure 2C).

Since hormones play an important role in cracking, we also focused on the pathways involved in hormone biosynthesis. Here, 85 genes related to receptors or transporters for abscisic acid, auxin and ethylene were found in the category of “plant hormone signal transduction” (Supplementary Table 3). Furthermore, many genes of pathways related to “cysteine and methionine metabolism” were associated with ethylene synthesis, including *SAMS* and *ACO*, which were significantly upregulated at 141 DASP compared to 96 DASP (Supplementary Tables 2, 3). Thus, the regulation of biosynthesis and signal transduction of ethylene were investigated. The gene encoding 1-aminocyclopropane-1-carboxylate oxidase (*ACO3*) was highly expressed at 141 DASP to regulate the process of ethylene biosynthesis (Figure 5B). The expression levels of ethylene receptor 2 (*ETR*), which is the first receptor for perceiving ethylene, were upregulated at 141 DASP compared to 96 DASP (Figure 5B), whereas the expression of the other negative ethylene signal transduction factor (*EBF1/2*) was downregulated. To further match ethylene changes during these stages, the ethylene content was detected. The ethylene production of aril was very low from 113 to 133 DAPS, and reached an obvious peak at 144 DAPS, followed by a decrease at 154 DASP (Figure 5C).

To further verify the RNA-Seq data, qPCR analysis was performed for 11 selected DEGs known to be related to aril cracking, including *TgFAR*, *TgPME*, *TgPG*, *TgPL*, *TgBLU*, *TgBAL*, *TgEXP, TgPOD, TgSAMS, TgACS*, and *TgACO* (Supplementary Figure 3B and Figure 5D). These unigenes were identified by comparisons of the two libraries, with the expression fold (log2 ratio ≥ 1.5) as the threshold. The RT-qPCR experiments showed that the transcripts’ expression of the most genes (except for *TgACS*) showed similar expression patterns to the results from RNA-Seq (Supplementary Figure 3 and Figure 5).

### Identification of Candidate Functional Genes and Regulation Genes for Seed Cracking in *T. grandis*

**FIGURE 6:**
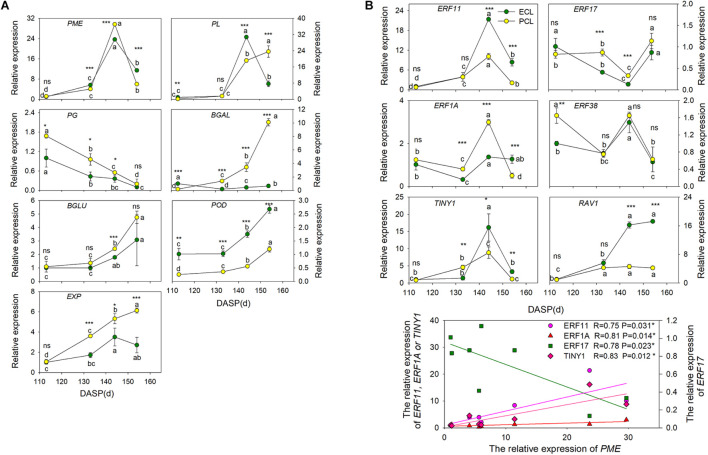
Changes in the expression of the cell wall modification-relevant genes and ethylene signal transcription factors of epidermal and parenchymatous cell layers in *T. grandis* during nut maturation. **(A)** Quantitative real-time PCR (RT-qPCR) of some cell wall modification-relevant genes. **(B)** Quantitative real-time PCR (RT-qPCR) of ethylene signal transcription factors selected by co-expression analysis. ECL, epidermal cell layer; PCL, parenchymatous cell layer. The expression levels were calculated relative to corresponding values at 113 DASP. Error bars represent standard error base on three biological replicates. Different lowercase letters indicate a significant difference at different growth stages (*P* < 0.05) by Duncan’s multiple range test. Asterisks denote significant differences using independent-samples *t*-test: *P < 0.05, **P < 0.01, ***P < 0.001, ^*ns*^*P* > 0.05. The solid line represents the best-fit linear regressions between PME and four ethylene signal transcription factors: *P < 0.05. *N* = 3.

To shed light on which genes play important roles in wax synthesis and cell wall modification pathways for *T. grandis* during seed cracking, the time-course transcriptome expression profiles of 7 unigenes (*EXP*, *PME*, β*-Gal*, β*-Glu*, *PG*, *PL*, and *POD*) of the epidermal and parenchymatous cell layers were analyzed from 113 to 154 DASP (Figure 6A). *EXP* was present both in the epidermal and parenchymatous cell layers, with a significant difference in the expression intensity between them after 133 DASP. Both the expression of *PME* in epidermal and parenchymatous cell layers showed an increasing trend from 113 to 144 DASP, followed by a decrease at 154 DASP. The expression of *PG* was decreased with the nut maturation. *PL* expression in the epidermal cell layer showed an increasing trend from 113 to 144 DASP, followed by a sharp decrease at 154 DASP, whereas the *PL* in the parenchymatous cell layer showed an increasing trend from 113 to 154 DASP (Figure 6A). The expression of β*-Glu*, β*-Gal* and *POD* in both epidermal and parenchymatous cell layers showed an increasing trend from 113 to 154 DASP.

To identify the key regulatory factors in the aril cracking process, co-expression network were constructed between different TFs and the cell modification genes based on the Pearson product-moment correlation coefficient. The correlation coefficient was calculated separately using the expression data at 96 and 141 DASP (0.9 for a positive correlation and −0.9 for a negative correlation). The resulting co-expression networks indicated that seven ethylene signal transcription factors (five *ERFs*, one *TINY* and one *RAV1*) were highly correlated since they were connected to most of their structural genes (Supplementary Figure 4A). The results of q-PCR revealed that the genes *ERF11*, *ERF1A*, and *ERF38* were significantly upregulated at 144 DASP compared to 133 DASP, whereas they showed a significant decrease of ERF17 and RAV1 (Supplementary Figure 4B).

The expression of *ERF11*, *TINY1*, and *ERF1A* in epidermal and parenchymatous cell layers sharply increased at 144 DASP compared to 133 DASP, whereas *ERF 17* had a contrasting trend (Figure 6B). Furthermore, there were significantly positive or negative correlations between *PME* and *ERFs* (*ERF11, ERF17*, and *ERF1A*) and *TINY1* (belonging to the AP2/ERF family).

### Effects of Ethylene or Ethylene Inhibitor 1-Methylcyclopropene on the Cracking of *T. grandis* Nuts

**FIGURE 7:**
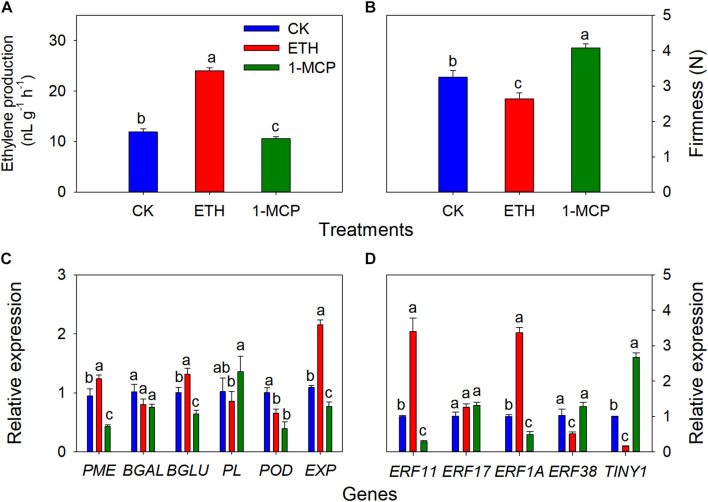
Changes in ethylene production of whole seeds, aril firmness, quantitative real-time PCR (RT-qPCR) of some cell wall modification-relevant genes, and five ethylene response factors in *T. grandis* at 9 days of treatments of 0 (CK), 3,000 μL L^− 1^ ethephon, or 200 nL L^− 1^1-MCP. **(A)** Ethylene production of whole seeds; **(B)** aril firmness; **(C)** RT-qPCR results of some cell wall modification-relevant genes; **(D)** RT-qPCR results of several ethylene response factors. Different lowercase letters indicate a significant difference at different growth stages by Duncan’s multiple range test (*P* < 0.05). *N* = 3.

To validate the role of ethylene on the cracking of *T. grandis* nuts, exogenous experiments involving ethephon, 1-MCP, and H_2_O (as control) were also performed. It showed that the ethylene production of whole seeds in the ETH treatments was significantly higher than in the CK and 1-MCP treatments (Figure 7A). Significant differences in firmness were found among CK, 1-MCP, and ETH treatments, where the firmness at 1-MCP was significantly higher than those in CK and ETH treatments (Figure 7B). The expression of *PME*, β*-Glu*, and *EXP* was significantly upregulated by ETH treatment, whereas the expression of these genes was significantly repressed by 1-MCP treatment (Figure 7C). *POD* expression in ETH and 1-MCP was significantly downregulated compared to CK. There were no significant differences in *PL* and β*-Gal* between CK and ETH treatments. *ERF11* and *ERF1A* expression was induced by ETH treatment but inhibited by 1-MCP (Figure 7D). However, the expression of *ERF38* and *TINY1* showed a contrasting trend. There was no significant difference in *ERF17* expression among the three treatments.

## Discussion

### Structural Changes in the Aril During *T. grandis* Seed Cracking

The material structure of the pericarp was softened by fruit ripening, as indicated by a decrease in fruit firmness. Yamaguchi et al. (2002) found that there was a significant correlation between firmness and cracking susceptibility. In the present study, aril firmness dropped sharply at 144 DASP (Figure 1A), indicating that *T. grandis* seed began senescence and ripening at 144 DASP. Previous research indicates that fruit cracking involves the cuticle layer and cell wall structures (Khadivi-Khub, 2015; Capel et al., 2017). The cuticle integrity during fruit growth has been proposed as one of the factors that influence the development of fruit cracking (Balbontín et al., 2014). In the present study, the intact cuticle layer structures at 133 DASP helped to maintain stable firmness (Figure 1A); then, the cuticle breakages at 144 DASP caused a loss in aril firmness. The occurrence of cracks in the surface cuticle is a symptom of fruit cracking, associated with greater enlarged rate in the aril than in fruit skin (pericarp) (Huang et al., 2004). Furthermore, the parenchymatous cell layer increased by 1.31-fold from 144 to 154 DASP, whereas the epidermal cell layer did not increase during this period. Thus, aril cracking in *T. grandis* could be attributed to an abruptly rapid growth of parenchymatous cells but a very slow epidermal cell growth that cannot offer enough space for parenchymatous cell growth.

Numerous studies have reported that changes in fruit firmness are directly related to cell wall degradation (Brummell, 2006; Bennett and Labavitch, 2008; Billy et al., 2008). The cell wall is a complex structure that consists of pectin, protein, cellulose, and hemicellulose (Goulao and Oliveira, 2008). Pectic substances hold plant cells together and their degradation results in a decrease in cell–cell cohesion and hardness (Liu et al., 2009; Paniagua et al., 2017). Many reports have shown that fruit softening is closely related to pectin solubilization and depolymerization (Rosli et al., 2004; Kurz et al., 2008). Pectic polymers WSP, CSP, or SSP represent the polyuronides loosely, ionically, or covalently bound to the wall, respectively (Vicente et al., 2007). WSP increased with maturity (Basanta et al., 2014), which may result in a weakening of the Ca^2+^ cross-linking of pectin (Ponce et al., 2010). It has been reported that CSP and SSP bear close relationships with fruit firmness (Brummell et al., 2004). Cell wall solubilization means displacement of polymers from one fraction to another due to the modifications of their structure (Jarvis, 1984). In the current study, the WSP content increased significantly from 133 to 154 DASP, whereas the CSP and SSP content significantly decreased during that period, which was consistent with the results from tomato fruit ripening that is usually accompanied by a shift in pectin from CSP and SSP to WSP (Gross and Wallner, 1979). In addition, both the content of 24KSF and 4KSF significantly decreased from 133 to 144 DASP (Figure 3B). Brummell et al. (2004) suggested that the reduction in firmness is generally accompanied by a decrease in insoluble pectin and hemicellulose, and an increase in soluble pectin content during the fruit ripening stages. In the present study, concomitant with cellular wall polysaccharide degradation, extensive lignification occurred in the edges between the aril and shell from 133 to 144 DASP, indicating a significant increase of lignin (Figures 2D, 3D). This was consistent with the fact that the pericarp contains areas of heavily lignified tissues at the site of separation during oilseed rape dehiscence (Meakin and Roberts, 1990). Thus, tentatively, aril cracking can be attributed to its cellular wall polysaccharide degradation and lignification.

### Possible Pathways Involved in Seed Cracking of *T. grandis*

Transcriptomes can produce large amounts of data, which is helpful for comprehensively and rapidly revealing the molecular mechanism of fruit cracking (Chen et al., 2019; Jiang H. K. et al., 2019; Wang et al., 2019a; Niu et al., 2020; Zhu et al., 2020); however, research using this technology has not previously been reported for seed cracking in *T. grandis*. Here, transcriptome sequencing was performed on 6 *T. grandis* aril samples at two different growth stages using RNA-Seq. The transcriptome sequences were *de novo* assembled into 121,185 transcripts, of which 78.41% were annotated (Supplementary Figure 1). To identify candidate structural and regulatory genes that contribute to *T. grandis* seed cracking during nut maturation, a comparison was performed, and 18,380 DEGs were identified between 96 and 141 DASP (Supplementary Figure 1). According to GO analysis, 5 GO terms in the top 20 enriched GO terms from “biological processes” were related to the cell wall (GO: 0071554; GO: 0071555; GO: 0044036; GO: 0042546; and GO: 0010383) in the 96 vs. 141 DASP comparisons (Figure 4A). Furthermore, KEGG enrichment analysis showed that the top 4 enriched pathways in the “metabolism” category were associated with cell wall metabolism (Figure 4B and Supplementary Table 3). Thus, the DEGs between 96 and 141 DASP may play important roles in seed cracking, which relates to cell wall metabolism.

Numerous studies have reported that some cell wall modification genes play roles in fruit cracking, including *EXP*, *PME*, *PL*, β*-Gal*, and β*-Glu* (Li et al., 2013; Khadivi-Khub, 2015; Chen et al., 2019; Niu et al., 2020). EXP has been shown to induce cell wall loosening to promote cell wall enlargement by disrupting non-covalent bonds between cellulose and hemicellulose (Sampedro and Cosgrove, 2005; Cosgrove, 2015; Barnes and Anderson, 2018). The expression of *AsEXP1* and *AsEXP3* is relatively high during cracking and ripening of cherimoya fruit (Shen et al., 2009). Previous studies have inferred that higher PG and PME enzymatic activity and transcript levels contribute to fruit cracking (Brummell and Harpster, 2001; Li et al., 2003; Lu and Lin, 2011; Li et al., 2014). However, downregulation of *LePG* gene expression slightly reduces the rate of tomato fruit cracking (Schuch et al., 1991). Inhibition of *Pp*β*-Gal* can delay peach fruit softening (Liu et al., 2018). Additionally, silencing the *SlPL* gene can enhance fruit firmness (Yang et al., 2017). Downregulation of β-Glu protein in strawberry can delay fruit maturation (Sara et al., 2019). Previous studies suggested that antisense inhibition of PME and PG activity in tomato reduced fruit cracking (Schuch et al., 1991; Brummell and Harpster, 2001). POD can activate several phenolics (such as monolignols) which will therefore cross-link, that may decrease the malleability of the cell wall in the pericarp, thereby inhibiting cell elongation (Lin and Kao, 2001; Andrews et al., 2002; Cordoba-Pedregosa et al., 2003). The POD activity in cracking-susceptible cultivars was significantly higher than that in cracking-resistant cultivars (Li et al., 2003; Cao et al., 2014; Yang et al., 2016; Xue et al., 2020). In the present research, transcriptome sequencing analysis found that the cell wall modification relevant genes, 11 *EXPs*, 4 *PMEs*, 10 β*-Gals*, 1 β*-Glu*, 1 *PL*, and 14 *PODs*, were significantly upregulated at 141 DASP compared to 96 DASP (Figure 5A), which was coincident with our RT-qPCR results (Figure 5B), suggesting that these genes may play important roles in the regulation of *T. grandis* seed cracking.

The structure and mechanical strength of the cuticle, which is an important part of the fruit pericarp with respect to cracking mechanisms (Domínguez et al., 2011; Petit et al., 2016), is related to the cutin matrix (Kunst and Samuels, 2003). Cuticle structure consists of a matrix of cutin polymers and cuticular waxes (Samuels et al., 2008; Domínguez et al., 2011). It has been reported that the cracking susceptibility between two contrasting cultivars could be related to the higher expression of genes involved in wax biosynthesis (Balbontín et al., 2014). In the present study, among the top 15 KEGG pathways of DEGs in 96 vs. 141 DASP, cutin, suberin, and wax biosynthesis (ko00073) was related to cuticle biosynthesis (Figure 4B). Major classes of aliphatic cuticular wax components include alkanes, ketones, fatty alcohols, fatty acids, and esters produced by acyl reduction and decarbonylation reactions of very long-chain fatty acids (VLCFA) in epidermal cells (Peschel et al., 2007). *CER1* is a core component of the complex involved in the decarbonylation pathway of wax biosynthesis, controlling very-long-chain alkane synthesis (Bourdenx et al., 2011; Bernard et al., 2012; Pascal et al., 2019). FAR is involved in the fatty acyl-CoA reductase pathway for citrus wax synthesis (Wang et al., 2016). In the present study, *CER1* and *FAR* (*FAR5*, *FAR3*, and *FAR2*) genes were downregulated at 141 DASP compared with 96 DASP (Supplementary Figure 3A). In addition, our real-time PCR result showed that the *FAR* gene significantly decreased after 113 DASP (Supplementary Figure 3B). These results indicate that the downregulation of these genes related to the biosynthesis of alkane and wax might play a role in fruit cracking.

The hormone balance in the peel has also been shown to influence fruit cracking (Khadivi-Khub, 2015). Notably, in our data, KEGG analysis revealed that the first top pathway was “plant hormone signal transduction” (ko04075), which is involved in the signal pathway of abscisic acid, auxin, and ethylene (Figure 4B and Supplementary Table 3). Furthermore, the genes enriched for the “cysteine and methionine metabolism” pathway (ko00270), which was related to ethylene biosynthesis, including *SAMS* and *ACO*, were upregulated at 141 DASP compared to 96 DASP (Figures 4B, 5B). These results were consistent with the common view that ethylene plays a critical role in fruit ripening (Balbontín et al., 2007). The expression of genes involved in the ethylene signaling pathway was further investigated. *ETR* and *EBF1/2* showed an opposite expression pattern during the seed cracking process (Figure 5B). It has been reported that the ETR2 receptors relieve the suppression of downstream responses by deactivating the negative regulator CTR1 when in the presence of ethylene; then, the signal can be transmitted via positive regulators EIN2 and EIN2/EIL1 (Ji and Guo, 2013). Silencing of the *SlBEF1/2* gene in tomato accelerates ripening, indicating that *BEF2* represses the ethylene response via the negative regulation of ethylene signal transduction (Yang et al., 2010). Thus, the upregulation of *ETR* and the downregulation of *BEF1/2* contribute to the ethylene response. In addition, the expression of the *ACO* gene reached its peak at 144 DASP, in parallel with the production of ethylene in aril (Figures 5C,D). It has been reported that there is a dramatic decrease in fruit hardness, which is accompanied by a peak in ethylene production (Costa et al., 2010). Similarly, an ethylene peak with a dramatic decrease in firmness was observed at 144 DASP (Figures 1, 5C). Those results suggested that the loss of aril firmness in *T. grandis* had a strong response to ethylene metabolism.

### Candidate Functional and Regulation Genes Involved in Seed Cracking of *T. grandis*

Fruit cracking is a complex physiological process controlled by numerous genes working together. To identify candidate structural and regulatory genes that contribute to *T. grandis* seed cracking during seed maturation, the expression of the cell wall modification-relevant genes was detected using RT-qPCR both in epidermal and parenchymatous cell layers from 113 to 154 DASP. Expression of the *FAR* gene in epidermal and parenchymatous cell layers significantly decreased at 113 DASP (Supplementary Figure 3B), indicating that the downregulation of *FAR* would decrease the production of cutin monomers and increase water permeability for seed cracking (Vogg et al., 2004). Interestingly, *POD* expression in the epidermal cell layer was significantly higher than that in the parenchymatous cell layer from 113 to 154 DASP, suggesting that it may hinder the elongation of the cell wall in epidermal cells, which will lead to aril cracking when elongation was accompanied by significantly higher expression of *EXP* at 133 DASP in parenchymatous cells (Figure 6A). Several cell wall degradation-related genes (*PME, PL*,β*-Gal*, and β*-Glu*) of epidermal and parenchymatous cell layers showed an increasing trend from 113 to 144 DAPS, suggesting that the degradation of cell wall polysaccharides, such as pectin, hemicellulose, and cellulose, and ultimately decreasing cell wall toughness, which is exhibited as aril cracking.

Furthermore, six ethylene signal factors (*TINY1*, *RAV1*, *ERF11*, *ERF17*, *ERF38*, and *ERF1A*) were identified by co-expression networks analysis between structural genes of cell wall modification and transcription factors (Supplementary Figure 4). RT-qPCR analysis showed that significant positive correlations were observed between *PME* and *TINY1*, *ERF1A*, and *ERF11* genes (*P* < 0.05), while there were negative correlations between *PME* and *ERF17*, indicating that these genes may be the candidate regulators of *PME* for *T. grandis* seed cracking (Figure 6B). The possible reason for the opposing trends of *ERF* expression during *T. grandis* seed development is that ERF proteins can both act as transcriptional activators or repressors (Fu et al., 2016). Li et al. (2013) identified that two ethylene response factors (*ZMdERF1* and *ZMdERF2*) can be induced when ethylene biosynthesis begins to increase, which are involved in the regulation of *ZMdPG1* for apple softening and dehiscence. A similar result was also obtained by Wang et al. (2007). In contrast, *CpERF9*, a transcriptional repressor of the cell wall modification genes (*CpPG5* and *CpPME1/2*), was found to control papaya fruit ripening and softening (Fu et al., 2016). *CIERF4* is a causative gene associated with fruit rind hardness and cracking resistance in watermelon (Liao et al., 2019). Here, our results suggested that *TINY1*, *ERF1A*, and *ERF11* may regulate *PME* during aril cracking process.

### The Effects on Ethylene or Ethylene Inhibitor 1-Methylcyclopropene on the Aril Cracking of *T. grandis*

Previous study showed that the decline of firmness in ‘Taishanzaoxia’ apple fruit was accelerated by ETH treatment, accompanied by a sharp increase of ethylene production, whereas the softening process was significantly limited associated with a inhibition of ethylene production under 1-MCP treatment (Li et al., 2013). In this study, our exogenous experiment showed that ethylene production of *T. grandis* seeds was significantly inhibited by 1-MCP, whereas it was induced by ETH treatment (Figure 7A). An opposite trend of firmness was also observed (Figure 7B). A long-term treatment with 1-MCP resulted in abnormal papaya fruit ripening, significantly inhibited the expression of *CpBEF1* (play important role in ethylene signaling transduction pathway) and several cell wall degradation-related genes, including *CpPGs, CpGAL, CpPMEs*, and *CpXYL* (Ding et al., 2019). Numerous studies showed that ethylene-signaling genes regulate the expression of cell wall degradation-related genes to affect softening in many fruits, for example, *AdERF9* involved in suppressing *AdXET5* promoter activity in kiwifruits (Yin et al., 2010), *MaERF11* repress the banana fruit ripening by binding to the promoters of *MaACO1*, *MaACS1*, and *MaEXPs* (Fan et al., 2016), *CpERF9* can directly bind to the *CpPG5* and *CpPME1/2* promoters (Fu et al., 2016). In the present study, the expression of *PME*, β*-Glu*, *EXP*, *ERF11*, and *ERF1A* was inhibited by 1-MCP, whereas the expression of these genes was induced by ETH treatment (Figures 7C,D). These results indicate that *ERF 11* and *ERF 1A* may control the *T. grandis* seed cracking process by regulating *PME* and *EXP.*

**FIGURE 8:**
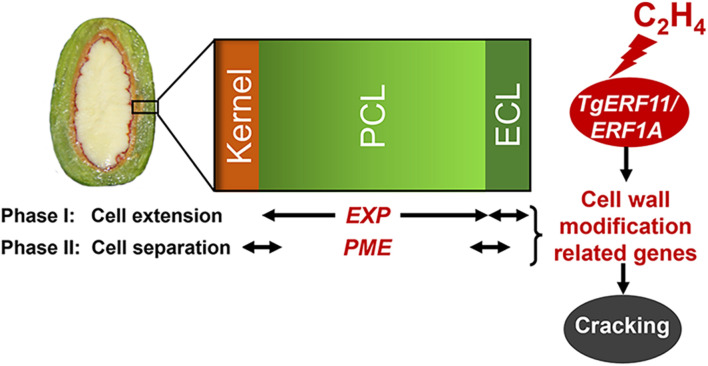
Proposed model for the regulation of cell wall modification genes (*EXP* and *PME*) by ethylene signal transcription factors (*ERF11* and *ERF1A*) in the presence of ethylene emissions during aril cracking in *T. grandis.*

Therefore, the cracking process of *T. grandi*s consisted of two phases (Figure 8): cell extension and cell separation of aril. The first phase was the rapid expansion of the epidermal cells and parenchymatous cell layer, especially a more rapid increase of *EXP* expression in parenchymatous cells than in the epidermis, resulting in cracks in the cuticle during the aril cracking process. In the second phase (cell separation), a clear separation layer was observed between the parenchymatous cell layer and the kernel, accompanied by a breakage between the epidermal and parenchymatous cell layers. During this period, both the epidermal and parenchymatous cell layers had significantly upregulated *PME* expression. Furthermore, ethylene was involved in the regulation of *EXP* and *PME* through ethylene signal transcription factors, such as *ERF11* and *EEF1A* (Figure 8).

## Conclusion

Cell wall composition and cell wall-relevant modification enzymes are major contributors to *T. grandis* seed cracking. The loss of firmness in *T. grandis* aril was associated with a shift in pectin from CSP and SSP to WSP, which was accompanied by a significant increase in the activity and gene expression of *PME*. In addition to *PME*, *EXPs* also play an important role during *T. grandis* seed cracking, especially in the parenchymatous cell layer. Moreover, by combining RNA transcriptome analysis and exogenous ethylene experiments, we demonstrated that ethylene production and ethylene signal transcription factor (ERF) may control the cracking process through the regulation of *PME* and *EXP*. In the future, further research should be conducted to determine how *ERF* functions to regulate related cell wall modification genes during seed cracking in *T. grandis*.

## Data Availability Statement

The data presented in the study are deposited in the NCBI’s SRA repository, accession number SRP338664.

## Author Contributions

LS and JW designed the work. YG and YH did running the experiments and data analysis and statistics. YG, YH, JyS, XM, JwS, ZZ, LS, and JW did the manuscript writing and revising. All authors contributed to the article and approved the submitted manuscript.

## Conflict of Interest

The authors declare that the research was conducted in the absence of any commercial or financial relationships that could be construed as a potential conflict of interest.

## Publisher’s Note

All claims expressed in this article are solely those of the authors and do not necessarily represent those of their affiliated organizations, or those of the publisher, the editors and the reviewers. Any product that may be evaluated in this article, or claim that may be made by its manufacturer, is not guaranteed or endorsed by the publisher.

## Footnotes

1
http://www.genome.jp/kegg/kegg2.html

2
http://primer3.ut.ee/

## Supplementary Material

Supplementary Figure 1 Characteristics of the homology search for *Torreya grandis* unigenes. **(A)** Venn diagrams of number of unigenes annotated by BLASTx with a cutoff *E*-value 1e^–05^ against protein databases. Numbers in the circles indicate the number of unigenes annotated by single or multiple databases. **(B)** *E*-value distribution of the top BLASTx hits against the Nr database. **(C)** Number of unigenes matching the 25 top species using BLASTx in the Nr database.

Supplementary Figure 2 Number of differentially expressed genes (DEGs) between aril at 96 and 141 DASP.

Supplementary Figure 3 Pathway of wax synthesis and the expression of the main gene involved in *T. grandis* during nut maturation. **(A)** Heat map of the differentially expressed transcripts involved in wax synthesis. **(B)** Quantitative real-time PCR (RT-qPCR) of the main gene FAR. Genes in red were significantly upregulated, and genes in green were significantly downregulated. Genes in black have no significant changes, and the standard is | log2FoldChange| > 1.5, *p*-value < 0.05, FPKM > 10. The expression level was homogenized by log10. CYP86, cytochrome P450 86B1; ACE/HTH, Protein HOTHEAD; CER1, protein ECERIFERUM 1; FAR, fatty acyl-CoA reductase. Different lowercase letters indicate a significant difference at different growth stages (*P* < 0.05). *N* = 3.

Supplementary Figure 4 Co-expression network between the candidate structural genes and transcription factors and the qPCR results of the six ethylene signal transduction genes. Co-expression networks between differentially expressed structural genes in the cell wall modification pathway and differentially expressed transcription factors (TFs) at 96 and 141 DASP. Dark green circles represent PMEs, yellow circles represent β-Glus, brown circles represent PLs, blue circles represent PGs, bright green circles represent PODs, and pink circles represent EXPs.

Supplementary Table 1 Quality of RNA-Seq libraries and the number of differentially expressed genes (DEGs) between arils at 96 and 141 DASP.

Supplementary Table 2 Summary of the total number of transcripts and the FPKM identified from different stages and replicates.

Supplementary Table 3 Summary of KEGG pathway functional enrichment analysis of DEGs between 96 and 141 DASP at the transcript level.

Supplementary Table 4 Sequences of specific primers used for the qPCR experiment.
