# Supplementary figures and images for: Acceleration of Aril Cracking by Ethylene in Torreya grandis During Nut Maturation
Source: Front Plant Sci. 2021 Oct 20;12:761139. doi: 10.3389/fpls.2021.761139 (PMC8565854; doi:10.3389/fpls.2021.761139)

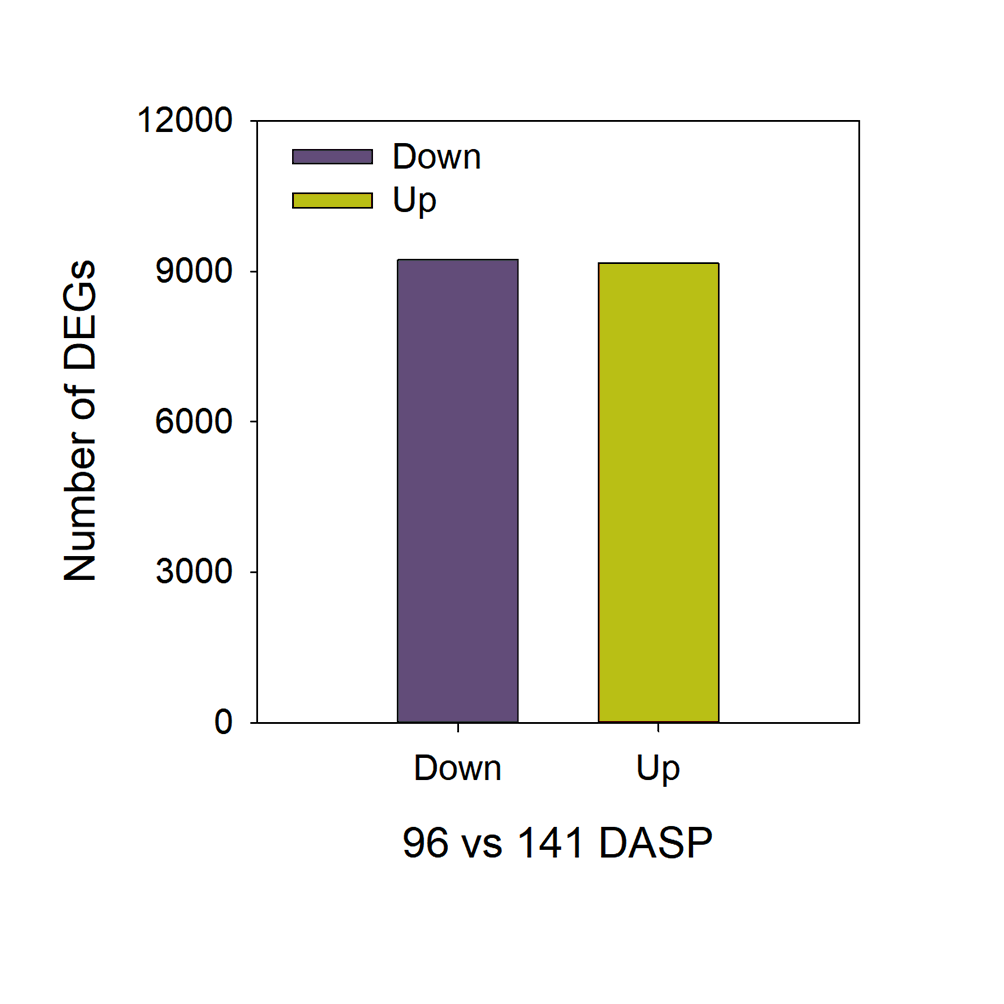

Supplement: Supplementary Figure 2 — Number of differentially expressed genes (DEGs) between aril at 96 and 141 DASP. [file Image_2.TIFF]

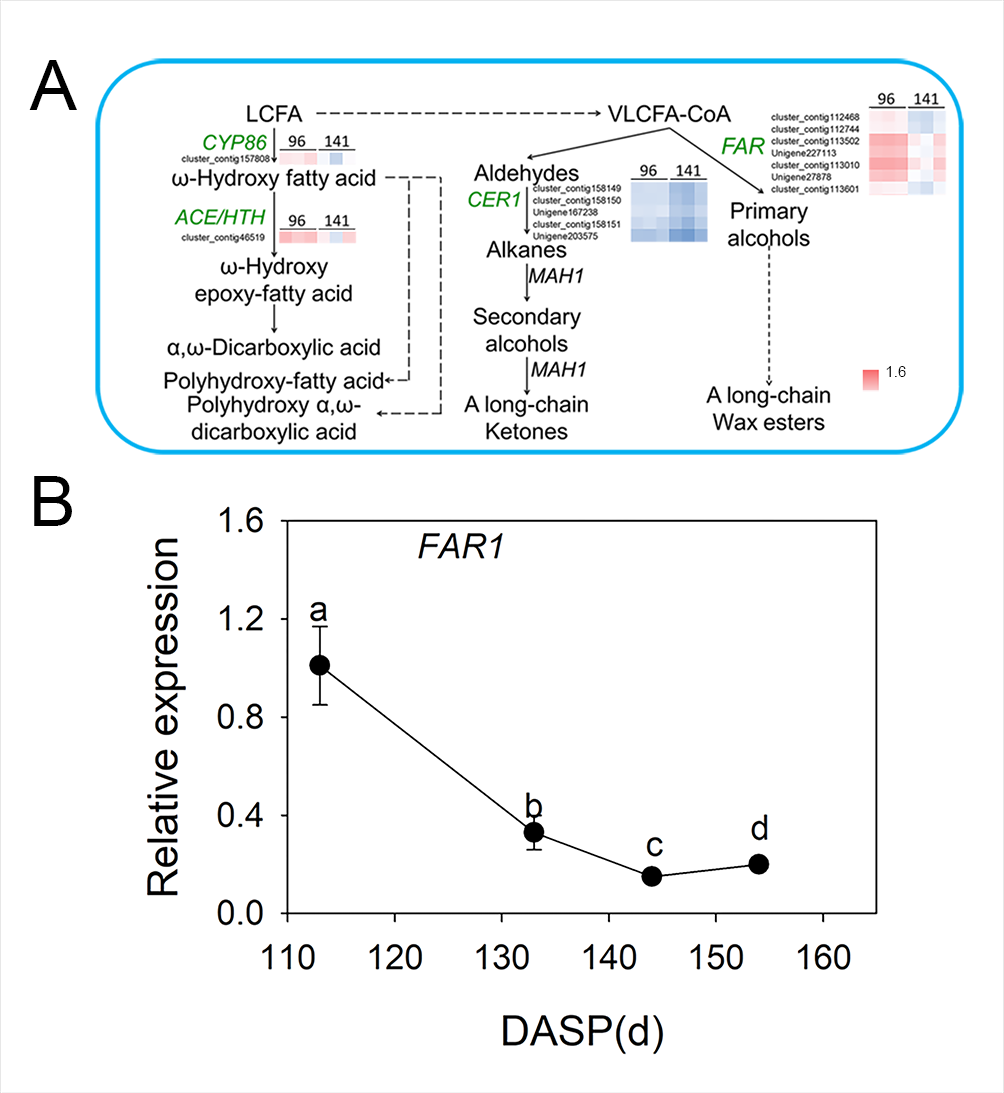

Supplement: Supplementary Figure 3 — Pathway of wax synthesis and the expression of the main gene involved in T. grandis during nut maturation. (A) Heat map of the differentially expressed transcripts involved in wax synthesis. (B) Quantitative real-time PCR (RT-qPCR) of the main gene FAR. Genes in red were significantly upregulated, and genes in green were significantly downregulated. Genes in black have no significant changes, and the standard is | log2FoldChange| > 1.5, p-value < 0.05, FPKM > 10. The expression level was homogenized by log10. CYP86, cytochrome P450 86B1; ACE/HTH, Protein HOTHEAD; CER1, protein ECERIFERUM 1; FAR, fatty acyl-CoA reductase. Different lowercase letters indicate a significant difference at different growth stages (P < 0.05). N = 3. [file Image_3.TIFF]

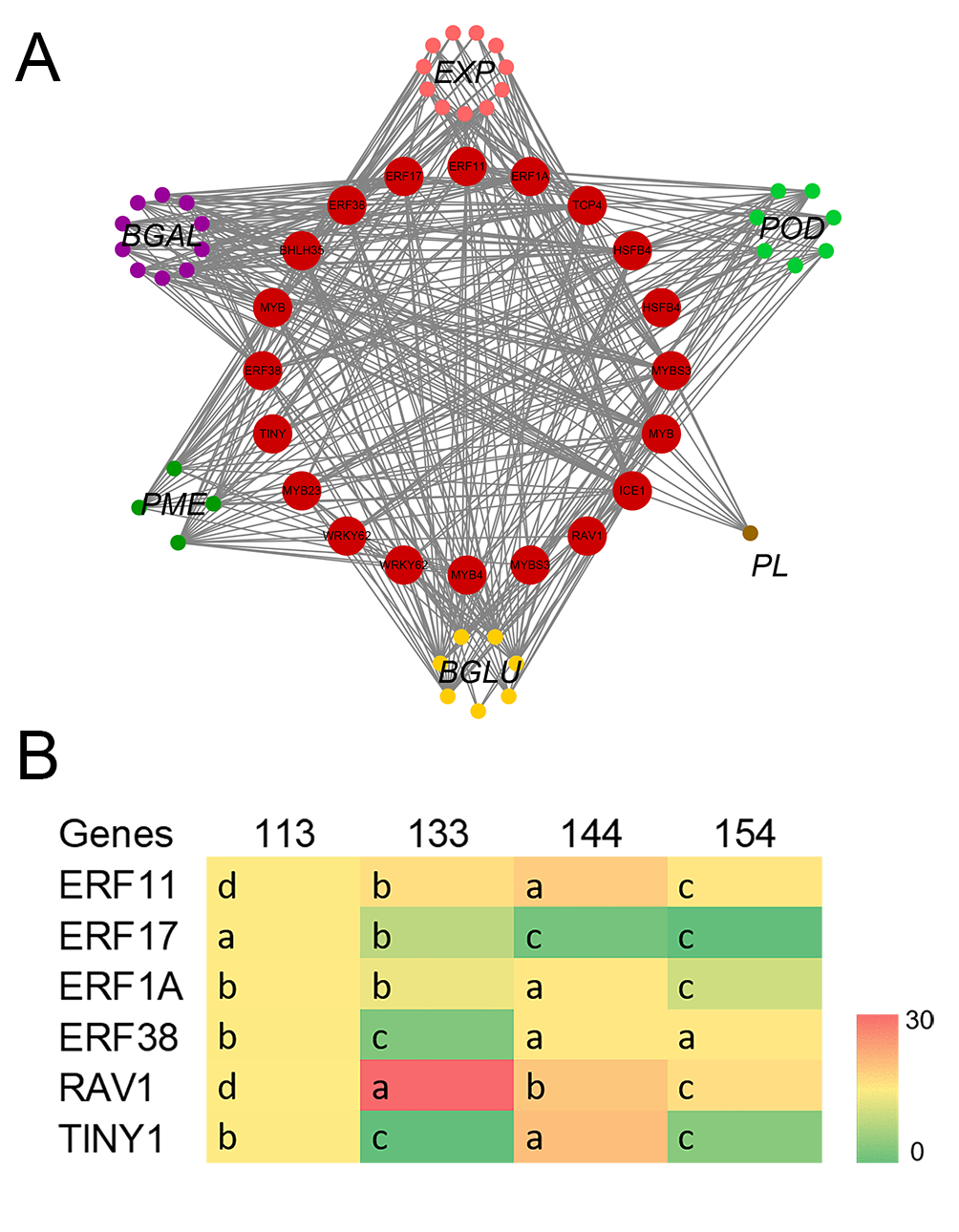

Supplement: Supplementary Figure 4 — Co-expression network between the candidate structural genes and transcription factors and the qPCR results of the six ethylene signal transduction genes. Co-expression networks between differentially expressed structural genes in the cell wall modification pathway and differentially expressed transcription factors (TFs) at 96 and 141 DASP. Dark green circles represent PMEs, yellow circles represent β-Glus, brown circles represent PLs, blue circles represent PGs, bright green circles represent PODs, and pink circles represent EXPs. [file Image_4.TIFF]
