# Supplementary material for: Acceleration of Aril Cracking by Ethylene in Torreya grandis During Nut Maturation
Source: Front Plant Sci. 2021 Oct 20;12:761139. doi: 10.3389/fpls.2021.761139 (PMC8565854; doi:10.3389/fpls.2021.761139)
Supplement: Supplementary Table 1 — Quality of RNA-Seq libraries and the number of differentially expressed genes (DEGs) between arils at 96 and 141 DASP. [file Table_1.DOCX]

Table S1 Quality of RNA-Seq libraries and the number of differentially expressed genes (DEGs) between arils at 96 and 141 DASP

| Library | Raw reads (bp) | Clean reads (bp) | Clean data (bp) | Q20 (%) | Q30 (%) |
| --- | --- | --- | --- | --- | --- |
| 96DASP-1 | 73,394,926 | 73,139,712 | 11,082,633,826 | 97.83 | 94.69 |
| 96DASP -2 | 69,092,536 | 68,848,922 | 10,432,972,936 | 97.8 | 94.6 |
| 96DASP -3 | 71,712,296 | 71,482,948 | 10,828,556,696 | 97.91 | 94.82 |
| 141DASP -1 | 74,303,576 | 73,982,058 | 11,219,839,976 | 97.28 | 93.43 |
| 141DASP -2 | 76,922,044 | 76,601,816 | 11,615,228,644 | 97.31 | 93.46 |
| 141DASP -3 | 71,656,518 | 71,371,292 | 10,820,134,218 | 97.38 | 93.64 |

Note: DASP, days after seed protrusion.
